# Supplementary material for: Deciphering acquired resistance mechanisms to sustained auxin-inducible protein degradation in cells and mice
Source: bioRxiv. 2025 Sep 23:2025.09.21.677607. Preprint. [Version 1] doi: 10.1101/2025.09.21.677607 (PMC12485725; doi:10.1101/2025.09.21.677607)
Supplement: Supplement 1 [file NIHPP2025.09.21.677607v1-supplement-1.pdf]

bioRxiv preprint doi: https://doi.org/10.1101/2025.09.21.677607; this version posted September 23, 2025. The copyright holder for this preprint (which was not certified by peer review) is the author/funder, who has granted bioRxiv a license to display the preprint in perpetuity. It is made available under aCC-BY 4.0 International license.

Supplementary Figure Legends

Supplementary Fig.1 Determine auxin-inducible degradation resistance in human CTCF-miniAID knock-in cell lines.

a. Schematic illustration of the Lentiviral OsTIR1$^{(F74G)}$ cassette, pCDH-MND-OsTIR1$^{(F74G)}$-P2A-Zeocin^{R}, and the miniAID-mClover3 tag knock-in design at the *CTCF* locus.

b. Immunoblot of Clone 27 parental and resistant cells with a titration of 5-Ph-IAA treatment for 24 hours. The CTCF immunoblot confirms that resistant cells were insensitive to auxin treatment even at higher doses. GAPDH was included as a loading control.

c. Quantitative PCR showing *OsTIR1*$^{(F74G)}$ expression in Clone 27 resistant cells is lower than that of parental cells. The expression shown is relative to GAPDH. *** p-value ≤ 0.001 calculated by unpaired *t*-test. N=3

d. Graph depicting increasing cell counts for Clones 3.2 and 17.2 after 25 days in culture with continuous 1 μM 5-Ph-IAA/ 100μg/mL Zeocin treatment. Cell counts taken from days 25 to 34 are shown.

e, f, g. Immunoblots from long-term 5-Ph-IAA culture of Clones 5, 20, and 26. All cultures were kept under continuous 1 μM 5-Ph-IAA/ 100 μg/mL Zeocin treatment. No T=no treatment. Lysates were blotted for CTCF, and GAPDH was included as a loading control. CTCF^{miniAIDmCLover3} expression did not escape auxin treatment in Clones 20 and 26.

Supplementary Fig. 2 Time-dependent tracing of miniAID mutation upon long-term auxin treatment.

a. Immunoblots from long-term 5-Ph-IAA culture of Clone 3.2 kept under continuous 1 μM 5-Ph-IAA/ 100 μg/mL Zeocin. Lysates were probed for CTCF and miniAID antibody, and GAPDH was included as a loading control. No T=no treatment. The auxin-resistant CTCF appeared around

bioRxiv preprint doi: https://doi.org/10.1101/2025.09.21.677607; this version posted September 23, 2025. The copyright holder for this preprint (which was not certified by peer review) is the author/funder, who has granted bioRxiv a license to display the preprint in perpetuity. It is made available under aCC-BY 4.0 International license.

day 25 of treatment. Auxin-resistant CTCF, which carried the P23S-AID mutation, was not detectable by immunoblotting with a miniAID antibody.

b and c. Sanger sequencing trace images from PCRs of Clone 3.2 parental and treated cells [cDNA (b) and genomic DNA (c)] of the miniAID sequence showed the C > T mutation in the miniAID tag of Clone 3.2 cells treated with 1 μM 5-Ph-IAA/ 100 μg/mL Zeocin for 34 days.

Supplementary Fig. 3 Time-dependent tracing of CTCF mutation upon long-term auxin treatment by immunoblot and mass spec analysis.

a. The full CTCF$^{\text{miniAID3-mClover3}}$ sequence representing mass spectrometry peptide coverage of the CTCF$^{\text{miniAID-mClover3}}$ protein in Clone 3.2 parental and Clones 3.2 and 17.2 resistant cells. Peptides that match the sequence with high confidence are shaded yellow and green.

b. Sequence illustration pinpointing the trypsin cleavage breakpoint observed by IP-MS in Clone 17.2 resistant cells. A red asterisk denotes the C > T mutation identified by RNA-seq of resistant cells.

c and d. Immunoblot from long-term 5-Ph-IAA culture of Clone 17.2 kept under continuous 1 μM 5-Ph-IAA/ 100 μg/mL Zeocin. Lysates were probed for CTCF and miniAID, and GAPDH was included as a loading control. Two replicates are shown. The auxin-resistant truncated-CTCF appeared around treatment day 18 of rep 1 and treatment day 25 of rep 2.

Supplementary Fig. 4 CTCF ChIP-seq of CTCF$^{\text{AID2}}$ clones expressing either CTCF$^{\text{WT-HA}}$ or CTCF$^{\text{Q666*-HA}}$

a. Immunoblots of CTCF$^{\text{AID2}}$ clones C5, C3.2, and C17.2 with doxycycline-inducible exogenous expression of CTCF$^{\text{WT-HA}}$ or CTCF$^{\text{Q666*-HA}}$. Cells were treated for 24 hours with 1μM 5-Ph-IAA to remove endogenous CTCF. After 6 hours of treatment, 0.05 μg/mL or 1.0 μg/mL doxycycline was added to CTCF$^{\text{AID2-WT-HA}}$ or CTCF$^{\text{AID2-Q666*-HA}}$ clones, respectively. The HA and CTCF antibodies

bioRxiv preprint doi: https://doi.org/10.1101/2025.09.21.677607; this version posted September 23, 2025. The copyright holder for this preprint (which was not certified by peer review) is the author/funder, who has granted bioRxiv a license to display the preprint in perpetuity. It is made available under aCC-BY 4.0 International license.

were used to assess exogenous CTCF protein expression. GAPDH antibody was used as a loading control.

b. Principal component analysis (PCA). PC1 showed 39.3% variance between the $\text{CTCF}^{\text{AID2-WT-HA}}$ or $\text{CTCF}^{\text{AID2-Q666*-HA}}$ clones. PC2 showed only 18.8% variance among the replicates.

c. Heatmap of CTCF ChIP-seq of $\text{CTCF}^{\text{AID2-WT-HA}}$ or $\text{CTCF}^{\text{AID2-Q666*-HA}}$ clones C5, C3.2, and C17.2 showed increased CTCF peak density at 7,727 CTCF binding peaks in $\text{CTCF}^{\text{AID2-WT-HA}}$ vs $\text{CTCF}^{\text{AID2-Q666*-HA}}$ clones (log2 FC > 1, FDR < 0.05). Peak density was reduced at only 59 CTCF binding peaks when comparing $\text{CTCF}^{\text{AID2-WT-HA}}$ vs $\text{CTCF}^{\text{AID2-Q666*-HA}}$ clones (log2 FC > 1, FDR < 0.05).

d. CTCF motif analysis of the 7,727 CTCF binding peaks with increased density in $\text{CTCF}^{\text{AID2-WT-HA}}$ vs $\text{CTCF}^{\text{AID2-Q666*-HA}}$ clones showed that the CTCF motifs were most enriched.

e. CTCF ChIP seq tracks of $\text{CTCF}^{\text{AID2-WT-HA}}$ and $\text{CTCF}^{\text{AID2-Q666*-HA}}$ clones (C3.2 and C17.2) at the *BLCAP* locus showed increased CTCF binding in $\text{CTCF}^{\text{AID2-Q666*-HA}}$ clones. No variance in binding was observed at most CTCF binding loci, as represented at the *ISYNA1* locus.

Supplementary Fig. 5 No auxin-inducible degradation resistance observed in human miniAID-RBM5 and MBNL1-miniAID knock-in cell lines.

a. Schematic diagram of the HA mini-AID knock-in design to the N-terminus of *RBM5* in SEM cells. Left-HA and Right-HA designate the homology arms (HA) that flank the targeted knock-in site. Immunoblots for RBM5 and HA showing that $^{\text{HA-miniAID}}\text{RBM5}$ remained sensitive to auxin throughout continuous 1 μM 5-Ph-IAA/ 100 μg/mL Zeocin treatment.

b. Schematic diagram of the HA mini-AID knock-in design to the C-terminus of *MBNL1* in SEM cells. Immunoblots for MBNL1 and HA showing that $\text{MBNL1}^{\text{HA-miniAID}}$ remained sensitive to auxin throughout continuous 1 μM 5-Ph-IAA/ 100 μg/mL Zeocin treatment.

bioRxiv preprint doi: https://doi.org/10.1101/2025.09.21.677607; this version posted September 23, 2025. The copyright holder for this preprint (which was not certified by peer review) is the author/funder, who has granted bioRxiv a license to display the preprint in perpetuity. It is made available under aCC-BY 4.0 International license.

Supplementary Fig. 6 Full characterization of Ctcf-miniAID knock-in mice and primary BCR-ABL B-ALL cells.

a. Successful knock-in was confirmed by genotyping PCR in founder mice. The genotyping PCR primers were combined to detect the 5' and 3' junctions of the miniAID. 5' F (red forward arrow) and R (blue arrow) primers, KI= 478bp; 3' F (black arrow) and R (red reverse arrow) primers, KI= 448bp; Homology arm primers outside the inserted miniAID sequences (red arrows): WT= 689bp, and KI= 893bp. A wild-type mouse was used as a negative control.

b. Successful germline transmission to the F1 progeny was confirmed by genotyping PCR. Primers were designed against the flanking regions of the miniAID cassette (red arrows). Tissues from wild-type and founder knock-in mouse 3F were used as negative and positive controls, respectively.

c. Immunoblotting of Ctcf-miniAID expression from tissues, including liver, spleen, and kidney of wild-type, heterozygous, and homozygous knock-in mice. All tissues expressed Ctcf-miniAID fusion protein with a molecular weight 7kDa higher than the wild-type Ctcf protein.

d. Simple schematic diagram representing BCR-ABL translocation and sensitivity to second-generation tyrosine kinase inhibitor. The translocation occurs between the ABL gene on chromosome 9 and the BCR gene on chromosome 22. Two translocation fusion proteins are observed: p210, a hallmark of chronic myeloid leukemia (CML), and p185, associated with ALL. TKI, tyrosine kinase inhibitor.

e. MTT assay was conducted in parental Ctcf$^{miniAID/miniAID}$ BCR-ABL B-ALL, following a 3-day treatment with Dasatinib in a dosage-dependent manner. K562 was used as a positive control cell line carrying the endogenous translocation of BCR-ABL, and SEM was used as a non-BCR-ABL negative control cell line, replication=3.

f. Bright field image demonstrating that Ctcf$^{miniAID/miniAID}$ BCR-ABL B-ALL cells are highly proliferative. Flow cytometry was conducted to show the high expression of B220 and the lack of

bioRxiv preprint doi: https://doi.org/10.1101/2025.09.21.677607; this version posted September 23, 2025. The copyright holder for this preprint (which was not certified by peer review) is the author/funder, who has granted bioRxiv a license to display the preprint in perpetuity. It is made available under aCC-BY 4.0 International license.

IgM in the homogenous populations. The eGFP channel indicated the expression of BCR-ABL from MSCV-BCR-ABL-IRES-eGFP.

g. Ctcf and miniAID immunoblot of Ctcf$^{\text{miniAID/miniAID}}$ BCR-ABL B-ALL cells showed Ctcf degradation after 6-hour 5-Ph-IAA treatment that was restored after auxin removal. Hsp70 was included as a loading control.

h. Schematic diagram illustrating CPA assay: Ctcf$^{\text{miniAID/miniAID}}$ BCR-ABL B-ALL cells were infected with Cas9 and sgRNA-CFP against the coding exons of *Ctcf*. Flow cytometry analysis of the CFP fluorescence percentage of the cells at day 2, day 5, and day 8 was traced.

i. CPA analysis showed time-dependent decreased cell proliferation after Ctcf knock-out. sgNT was included as a negative control, and sgMyc was included as a positive control.

Supplementary Fig. 7 Establishment of the *in vivo* Ctcf$^{\text{miniAID/miniAID}}$ BCR-ABL B-ALL mouse model and characterization of acquired auxin resistance following long-term *in vivo* 5-Ph-IAA treatment.

a. MTT assay showed Ctcf$^{\text{miniAID/miniAID}}$ BCR-ABL B-ALL cells after long-term 5-Ph-IAA treatment were still sensitive to Dasatinib, similar to parental cells. SEM cells were included as a negative control.

b. Immunoblotting of Ctcf in Ctcf$^{\text{miniAID/miniAID}}$ BCR-ABL B-ALL cells treated long-term (day 29) with 5-Ph-IAA showed acquired auxin resistance. HSC70 was included as a loading control.

c. Schematic diagram showing *in vivo* model establishment: 100K cells were injected through the tail vein. Weekly peripheral blood was collected for flow analysis until the endpoint.

d. Flow analysis of weekly leukemia cell percentage (GFP+ cells) from peripheral blood.

e. Image showing spleen size after Ctcf$^{\text{miniAID/miniAID}}$ BCR-ABL B-ALL cell injection compared with the negative controls.

f. Spleen weight/ body weight of the Ctcf$^{\text{miniAID/miniAID}}$ BCR-ABL B-ALL cell-injected mice compared with negative controls.

bioRxiv preprint doi: https://doi.org/10.1101/2025.09.21.677607; this version posted September 23, 2025. The copyright holder for this preprint (which was not certified by peer review) is the author/funder, who has granted bioRxiv a license to display the preprint in perpetuity. It is made available under aCC-BY 4.0 International license.

g. Spleen H&E staining of the control and Ctcf$^{miniAID/miniAID}$ BCR-ABL B-ALL cell-injected mice.

h. Spleen eGFP staining of the control and Ctcf$^{miniAID/miniAID}$ BCR-ABL B-ALL cell-injected mice.

i. After Ctcf$^{miniAID/miniAID}$ BCR-ABL-B-All cells were infected with luciferase-YFP, immunoblotting of Ctcf, miniAID confirmed Ctcf-miniAID fusion protein degradation after 6-hour 5-Ph-IAA treatment before *in vivo* transplantation.
